# Recent advances in the design of cathode materials for Li-ion batteries

**DOI:** 10.1039/d0ra03314f

**Published:** 2020-06-08

**Authors:** Nourhan Mohamed, Nageh K. Allam

**Affiliations:** Energy Materials Laboratory, School of Sciences and Engineering, The American University in Cairo New Cairo 11835 Egypt nageh.allam@aucegypt.edu

## Abstract

The Li-ion battery (LIB) industry has rapidly developed and dominates the market of electric vehicles and portable electronic devices. Special attention is devoted to achieving higher power and energy densities, along with enhancing safety and reducing cost. Therefore, critical insights should be made on the understanding of the behavior of the components of LIBs under working conditions in order to direct future research and development. The present review discusses the literature on the properties and limitations of different cathode materials for LIBs, including layered transition metal oxides, spinels, and polyanionic positive electrode materials, with critical insights on the structural, thermal, and electrochemical changes that take place during cycling. Besides, the strategies and techniques capable of overcoming current limitations are highlighted.

## Abstract 2

It is crucial to design new innovative materials to boost the performance of Li-ion batteries.

## Graphical abstract

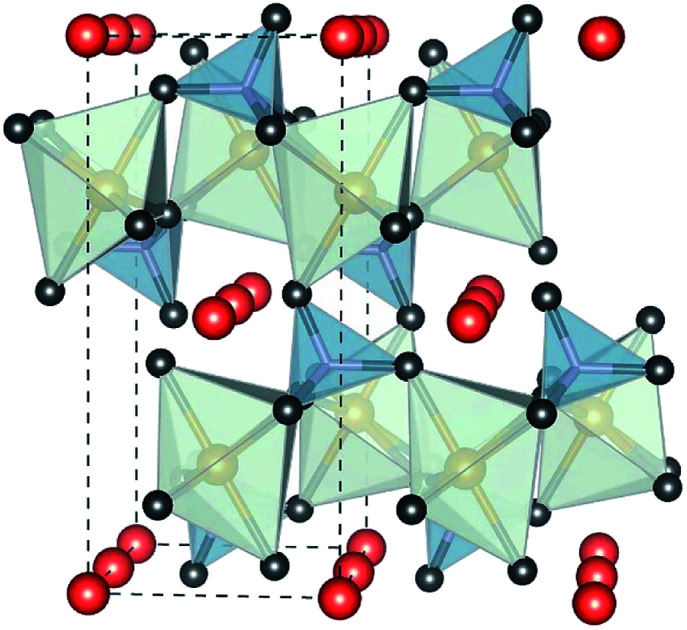

## 1 Introduction

The pressing demand to introduce alternative energy sources to fossil fuels and minimize CO_2_ emissions generates considerable recent research interest in the development of renewables along with energy storage systems. Shortly, the Earth is expected to run out of petroleum resources as they are being depleted at a fast rate. In addition, avoiding petroleum resources is necessary because of their negative environmental impacts, such as the water and air pollution that led to global warming problems. At the global level, renewable sources show a great theoretical ability to cover the whole human need for energy. For instance, Earth receives 1.2 × 10^5^ TW of solar power, which can cover the annual worldwide need for energy in almost one hour. Despite the current limitations in the properties of materials that lead to inefficient collection and conversion of light, the primary current focus is on how to progress rapidly towards highly functional materials to satisfy the ever-increasing energy demand.

**Fig. 1:**
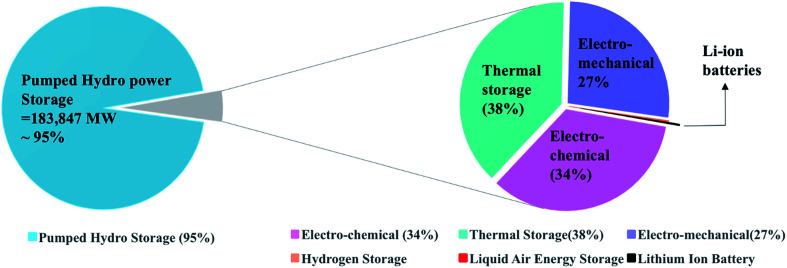
Worldwide energy storage projects contribution to the grid applications. Data is extracted from US-DOE.^3^

Energy storage technologies are divided into stationary and portable systems; both are of great importance, and each type is at a different stage of development. In multiple stationary applications, grid-connected energy storage systems have been deployed to facilitate the integration of renewable power sources due to their variable nature. Energy storage systems are connected to the grid to provide sustainability by storing energy during the low-energy demand time and supply it during the high-energy demand time. Nowadays, the global energy storage is dominated by one technology, the pumped hydropower, for grid applications, which accounts for 95% of the worldwide energy storage. The rest 5% comprise mainly of thermal, mechanical, and electrochemical storage. In comparison, a project of Li-ion batteries contributes 24 MW that is as low as 0.01% of the overall storage, as shown in Fig. 1.

Advances in renewable energy require modernization of the electricity storage systems, including electrochemical capacitors, lead-acid batteries, high-temperature sodium batteries, lithium-ion batteries, and redox flow batteries. In this review, we focus mainly on Li-ion batteries because of their superior performance in comparison with other conventional battery types. Li-ion batteries dominate the portable devices industry due to their prime properties such as high-energy-density, high charge/discharge rate, low maintenance, and long-lifetime, which make them the most suitable candidates for a plethora of applications. Therefore, they met the market requirements, and their sales increased significantly between 2008 and 2014 from 0.1 to 22.5 billion dollars in the electric vehicles sector and from 9.1 to 31 billion dollars in the total market.^1,2^

Li-batteries have been widely adopted because Li is the lightest metal (6.94 g mol^−1^) with the smallest radius that can provide high gravimetric and volumetric capacity, leading to a significant weight and volume reduction of the battery. Furthermore, Li exhibits the lowest reduction potential (−3.04 V *versus* SHE), allowing the cell to deliver the highest possible potential resulting in a decrease in the number of cells required to operate a device. However, several safety problems caused the withdrawal of Li metal batteries from the market after some catastrophic accidents because of the high reactivity of the Li metal and the growth of Li dendrite, which results in short circuits. Therefore, Li metal anode was replaced by safer alternatives such as intercalation, alloying, and conversion materials. Consequently, much research has focused on the chemical, thermal, and structural stability of the materials for Li-ion batteries and how to introduce practical solutions to their well-recognized safety problems. These problems are related to the reactions that can take place under extreme conditions such as high over-potential, electrolyte decomposition, SEI decomposition, cathode material decomposition, and generation of flammable gases. The safety stands as an obstacle to the introduction of new high energy cathode materials because they work outside the stability window of the available electrolytes. Improving the performance, along with the cost and safety, are the key factors to expand the Li-ion battery applications significantly. A significant interest in Li-ion batteries is given to the cathode materials and how to improve its electrochemical performance along with preserving the mechanical, electrochemical, and chemical stability of the materials upon cycling. Because the performance of the battery, including cell potential, energy density, power density, lifetime, and safety in addition to cost, depends to a great extent on the cathode material. For instance, high energy can be obtained from a battery by increasing the intercalation voltage (cathode material type) or the amount of Li^+^ that can participate in the electrochemical reaction (capacity). In this review paper, we focus on different types of cathode materials and discuss their electrochemical properties considering their structure and morphology as well as thermal and electrochemical performance.

## 2 Li-ion batteries (LIBs)

A Li-ion module consists of several cells connected into packs to deliver the required voltage, power, and energy. LIBs convert chemical energy into electricity *via* a reversible electrochemical reaction. They consist mainly of anode and cathode, which are electronically isolated by an electrolyte. The anode is the negative electrode, which is oxidized during the electrochemical reaction to donating electrons to the external circuit. The cathode is the positive electrode, which is reduced during the electrochemical reaction and accepts electrons from the external circuit. The electrolyte is a solid or a liquid medium that allows the Li ions to move between the anode and cathode and force the electrons to move through the external circuit. Therefore, electrolyte should exhibit high ionic and low electronic conductivity. Moreover, the Fermi level of the anode should be at lower energy than the LUMO of the electrolyte to prevent the electrolyte reduction, while the Fermi level of the cathode should be at higher energy than the HUMO of the electrolyte to prevent the electrolyte oxidation. During the first charge, the electrolyte undergoes a reduction reaction at the surface of the anode forming an irreversible passive layer, known as solid electrolyte interphase (SEI). It is a complicated layer composed of organic and inorganic components that prevent the further decomposition of the electrolyte upon cycling, but it results in an irreversible capacity loss. The performance of the battery is dependent on the morphology, composition, and thickness of this layer.^4^ If this protective layer is damaged, the decomposition of the electrolyte will continue again, leading to fast heating and undesirable chemical reactions that are hazardous. There are several reported mechanisms for the formation and stabilization of the SEI on the surface of both anode and cathode.^5–8^

## 3 Challenges and trend in cathode materials for Li-ion batteries

The development of the cathode materials for Li-ion batteries remains challenging because the existing materials such as layered transition metals oxides, olivines, or spinel all show upsides and downsides. For example, the layered oxides such as LiCoO_2_ suffers from the instability that limits their potential window and capacity. Moreover, the toxicity and the high production cost of the Co-based materials are undesirable. On the other hand, Li-rich layered oxides (Li_1+*x*_M_1−*x*_O_2_), where M is a mixture of transition metals (Ni, Mn, and Co), is promising as they can work at high discharge voltages of >4.5 V and deliver high specific capacities. However, they suffer from large voltage decay during cycling and high irreversible capacity loss at the first cycles, which limit their use. Moreover, olivine materials such as LiFePO_4_ are competitive candidates surpassing the stability of layered oxides at elevated temperatures due to their high thermal and structural stability. However, their low electronic and ionic conductivity stand as an obstacle to expand their use for high energy Li-ion batteries. Several steps forward have been made in the development of cathode materials for Li-ion batteries to overcome current challenges. First, investigating the changes in the electrochemical performance inside the cathode during cycling will lead to understanding the origin of the malfunction of the materials under working conditions. The strategies considered to improve these materials should address the problems related to the electrochemical performance side by side with the safety and cost issues. These strategies mainly include designing nanostructured materials with controlled morphology, introducing artificial defects in the nanostructured materials, using nanostructured complexes by doping or surface coating, and surface modification that is indispensable to minimize the undesirable side reaction at the electrolyte–cathode interface. Those are critical factors in jeopardizing the electrode performance. Also, there is a great interest in identifying new cathode materials that can fulfill the required balance between the performance and environmental requirements. In this review, we compare the different cathode materials that have been widely used in Li-ion batteries and analyze their performance.

## 4 Layered transition metal oxides positive electrode materials

**Fig. 2:**
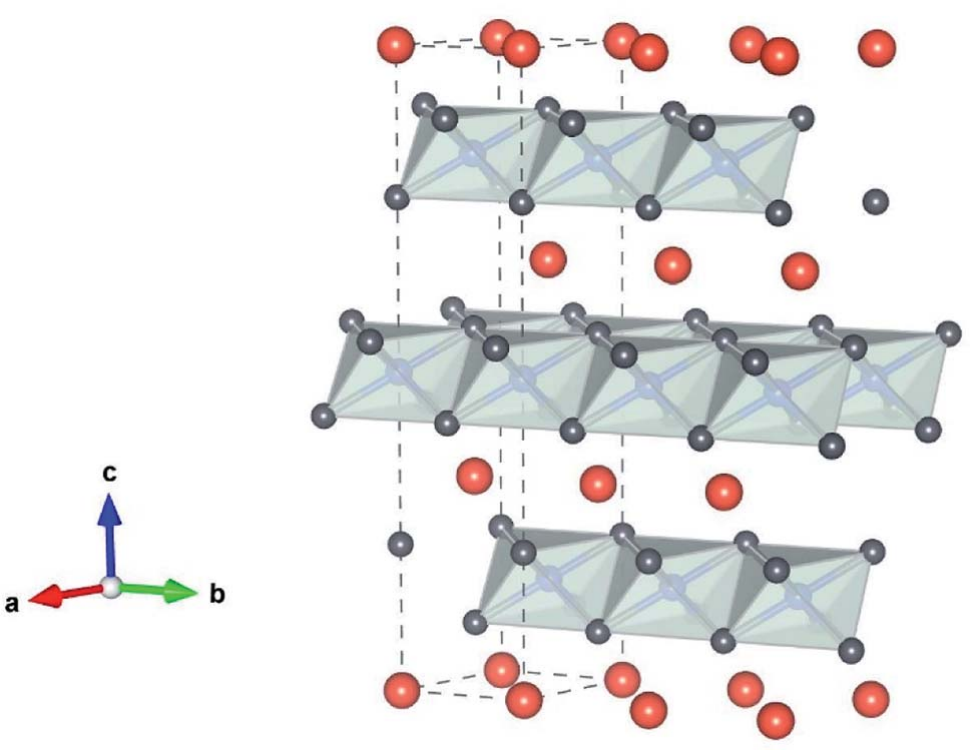
The crystallographic framework of the layered LiMO_2_ (M is Co or Ni) consisting of MO_6_ octahedra (gray) and Li^+^ (orange) “by VESTA”.

The reason for the continued attention to transition metal oxides LiMO_2_ (M = Co, Mn, Ni) as electrode materials is their high Li^+^ mobility in 2-dimensional space, thanks to its layered structure, as shown in Fig. 2. The crystallographic framework of Layered LiMO_2_ (O3 type) is α-NaFeO_2_-like structure, which consists of a cubic close-packed oxygen array occupying the 6c sites, where Li and M ions occupy the 3a and 3b sites, respectively. M and Li ions occupy alternate layers in the octahedral sites along the (111) plane forming ABC stacking sequence. The cell parameters of LiMO_2_ (M = Co and Ni) are shown in Table 1.

**Table 1:** Cell parameters of LiMO_2_ (M is Co or Ni)

LiMO_2_	*a* (Å)	*c* (Å)	*c*/*a*	*V* (Å^3^)	Ref.
LiCoO_2_	2.8157(3)	14.0517(3)	4.99	96.48(3)	9
LiNiO_2_	2.8898(2)	14.2212(2)	4.92	102.85(6)	

### 4.1 LiCoO_2_

**Fig. 3:**
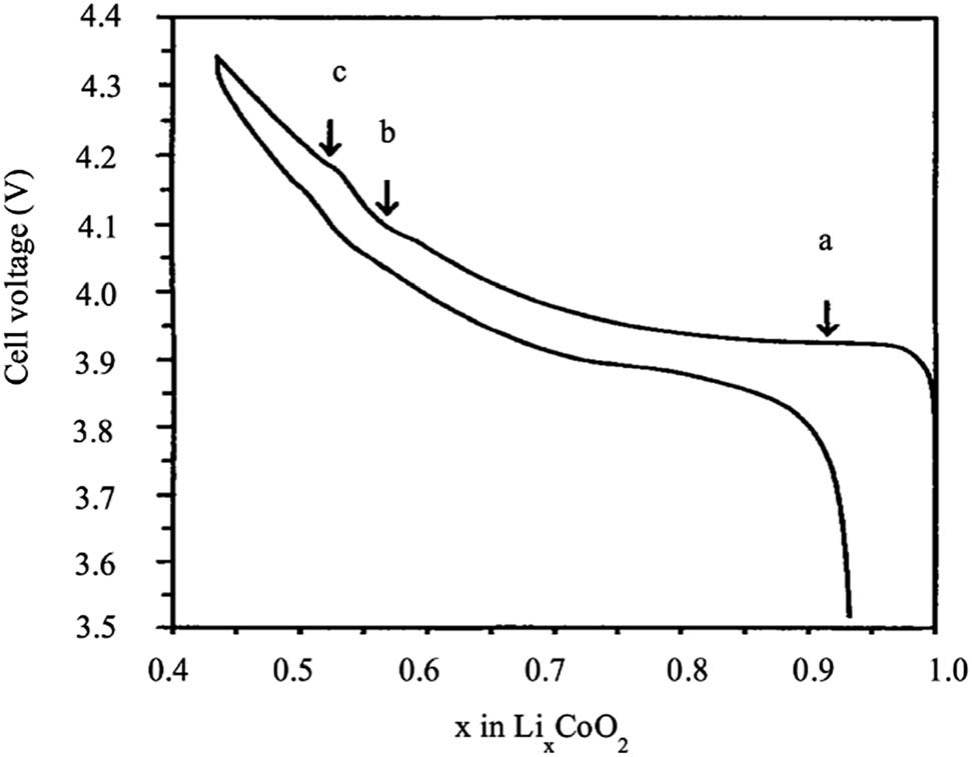
Cell voltage of Li_*x*_CoO_2_ as a function of *x*.^12^

LiCoO_2_ represents a significant advance in the history of rechargeable Li-ion batteries, as it was the first commercialized positive electrode material by Sony in 1991. Sony combined the LiCoO_2_ cathode and carbon anode to produce the first successful rechargeable Li-ion battery. From the electrochemistry point of view, Li_*x*_CoO_2_ is a very attractive material because it exhibits a high theoretical capacity of 274 mA h g^−1^ and high energy density. However, its performance is strongly affected by structural and thermal instability at high voltage, and high temperature, as well as fast capacity, fade at the high current rate or deep cycling. During charging, several phase transitions take place at different stages of the de-intercalation of Li^+^. Fig. 3 shows a typical charge/discharge behavior of Li_*x*_CoO_2_ as a function of the amount of *x*. The large plateau at 3.93 V *vs.* Li^+^/Li^0^ corresponds to a first-order transition (H1 ↔ H2). The coexistence of two phases between the lithium concentrations *x* = 0.75 and 0.93 is accompanied by the transition from a semiconductor to the metal, which facilitates the charge transfer. In the *x* range between 0.75 and 0.25, two order/disorder transitions were observed from hexagonal to monoclinic, then from monoclinic to hexagonal, which correspond to the plateaus *b* and *c*. Therefore, almost one-half of the lithium ions can be cycled reversibly in and out of the compound corresponding to the composition range between *x* = 1 and 0.5. Consequently, a practical specific capacity of 140 mA h g^−1^ is obtained at a charging voltage of 4.2 V *vs.* Li/Li^+^ to avoid the structural rearrangement of the material.^10,11^

It was traditionally accepted that the positive charge decrease by Li ions removal from Li_*x*_CoO_2_ is compensated by the increased charge of transition metal ions. At the same time, O^2−^ electronic state is fixed to keep the electro-neutrality of the material. But sensitive techniques to the oxidation state, spin state, bond covalence, and the electronic structures such as EELS and XAS suggest that both oxygen and transition metal ions experience an electronic change, and both participate to the electrochemical reaction. The oxygen K-edge XAS for Li_*x*_CoO_2_ shows significant change with respect to Li-ion content compared to the Co L_II,III_-edge XAS, suggesting that the electronic structure of the oxygen ions is more influenced than Co ions by the Li^+^ removal. In addition, the capacity fading upon cycling is explained with the change in the Co–O covalence that changes with the Li content as a result of the local structural distortion of CoO_6_ octahedra that accompanies oxygen and cobalt orbitals re-hybridization.^13^

**Fig. 4:**
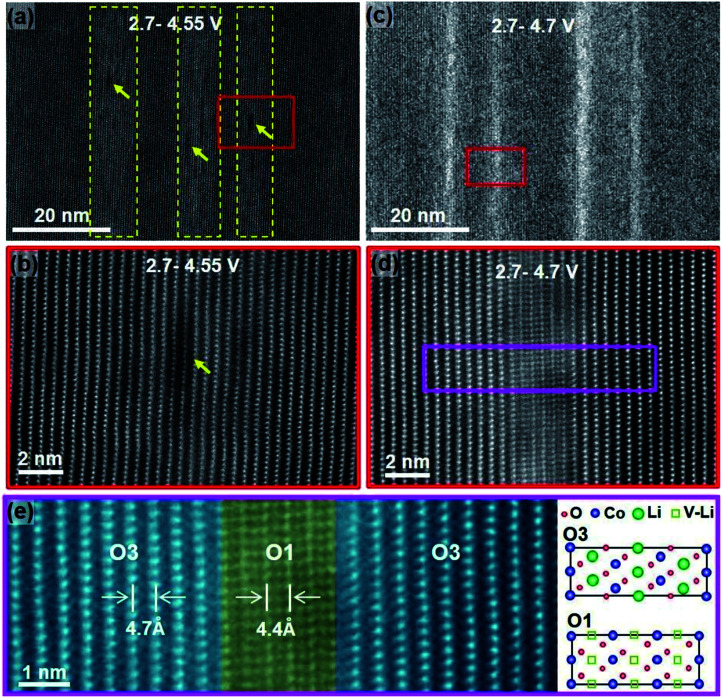
STEM-HAADF images showing the degradation induced from cycling up to high cutoff voltages for 50 cycles. (a) STEM-HAADF image showing the bright stripes (highlighted by dashed yellow frames) and the dark pits (yellow arrows) on sample cycled between 2.7–4.5 V, (b) High-resolution lattice image to show lattice images in (a), (c) STEM-HAADF image showing the bright stripes formed in the sample cycled between 2.7–4.7 V, (d) lattice image from the region marked in (c), and (e) enlarged lattice image from the pink region in (d).^14^

**Fig. 5:**
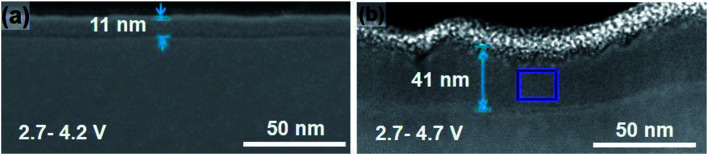
Effect of high charge cutoff voltage on the thickness of the surface degradation layer.^14^

Degradation has a significant contribution to the performance decay of LiCoO_2_. Bulk structure degradation of LiCoO_2_ is the main reason for capacity loss at high voltage. Jiang *et al.* observed that by increasing the cutoff potential from 4.2 to 4.7 V, Li concentration was significantly decreased in the bulk.^14^ Besides, the samples with low Li content suffered from thermal instability when cycled to 4.7 V. The STEM-HAADF images, Fig. 4, show bright strips, where in the middle, the O1 phase is identified, and the density of these bright strips increases with increasing the cutoff voltage and the number of cycles. This reveals that there are uneven lithiation and de-lithiation behaviors in the bulk material. Besides structure degradation, interfacial degradation is one of the mechanisms assigned to the failure of layered cathode materials. It originates from the surface phase transformation layer and the cathode electrolyte interface (CEI) layer. The surface degradation is enhanced by increasing the cutoff potential. Fig. 5 shows that the thickness of the degradation layer after 50 cycles is about four times higher in the samples cycled to a cutoff voltage of 4.7 V compared to that cycled to 4.2 V.

Tremendous efforts have been devoted to improving the functional performance of Li_1−*x*_CoO_2_ at high voltage and elevated temperatures. Surface coating, doping with foreign elements, and synthesis by wet chemistry methods such as sol–gel are traditional strategies that have been explored to improve the materials' properties. There are several methods for surface coating, including wet chemistry (sol–gel), chemical polymerization routes, and deposition techniques (sputtering, pulsed laser deposition, atomic layer deposition, chemical vapor deposition).^15^ The properties of the coated material depend on the homogeneity and thickness of the coating layer. Insulator metals oxides, such as AlO_2_, ZrO_2_, and AlPO_4_, show outstanding improved performance in terms of capacity retention and rate capability at high voltage compared to the bare LiCoO_2_. The enhanced electrochemical performance is attributed to (1) the suppression of cobalt dissolution in the electrolyte, (2) the prevention of the direct contact between the cathode and the liquid electrolyte by the inactive coating layer,^16^ and (3) cation diffusion to form Li–M–O (M = Al, Co) on the surface during calcination that can suppress the phase transitions and hence decrease the capacity fading especially at high potentials.^17^ Recently, Yano *et al.*^18^ synthesized 0.5 wt% AlO_2_-coated LiCoO_2_ by the sol–gel method. It shows electrochemical activity at potential > 4.2 V *vs.* Li/Li^+^ and an initial capacity of 247 mA h g^−1^ at the first cycle and preserved ∼82.6% of its initial capacity after 20 cycles. The improved performance is attributed to the formation of a solid solution surface phase of LiAlO_2_ and LiCoO_2_ by the diffusion during the calcination after the sol–gel coating. The coated LiCoO_2_ shows very small phase transition upon cycling as Al^3+^ is electrochemically inactive and shares the octahedral sites with Co ions in the MO_6_, resulting in a difficultly for both Li and O ions to leave the LiAlO_2_ framework and thereby preserving the surface structure. Table 2 lists several examples of the improved electrochemical performance at high cutoff voltage by coating. Moreover, conductive oxides or Li ion conductors that show low interfacial resistance and maintain high ionic conductivity such as Al–ZnO, lithium phosphorus oxynitride and lithium tungsten oxide (LWO) are excellent alternatives to the insulator metal oxide coating.^19,20^

**Table 2:** The performance of coated LiCoO_2_ by different materials and different techniques

Surface coating material	Coating technique	Electrochemical performance	Ref.
Al_2_O_3_	Vapor-assisted hydrolysis	165.5 mA h g^−1^ @ 360 mA g^−1^, voltage range (3–4.5 V *vs.* Li/Li^+^), capacity retention 98.6% (180 cycle)	21
Al_2_O_3_	Atomic layer deposition	175.72 voltage range (2.5–4.5 V *vs.* Li/Li^+^), capacity retention 94.9% (50 cycle)	22
AlW_*x*_F_*y*_	Atomic layer deposition	180.62 voltage range (2.5–4.5 V *vs.* Li/Li^+^), capacity retention 94.99% (50 cycle)	
AlF_3_	2 ALD (LCO-2% MWCNT-5% NCF) paper electrode	216 mA h g^−1^, cutoff voltage 4.7 V, capacity retention (75.7%, 70% after 100 and 160 cycle)	23
(1.0 wt%) AlPO_4_	Solution	233 mA h g^−1^ (cutoff voltage = 4.8), capacity retention ∼64% (50 cycle)	24
(2 wt%) AlPO_4_	Freeze drying method	∼210 mA h g^−1^ (2.75–4.55 V), capacity retention 86.1% (50 cycle)	19
(2 wt%) LiAlPO_3.93_F_1.07_	Freeze drying method	206 mA h g^−1^ (2.75–4.55 V), capacity retention 91.7% (50 cycle)	
(20 nm) Al_2_O_3_-doped ZnO	Magnetron sputtering coating	193 mA h g^−1^ voltage range (3–4.5), capacity retention 90% (150 cycle)	25
(2 wt%) CeO_2_	Sol–gel	138.7 mA h g^−1^ (3–4.4 V), ∼89% (50 cycle)	26
(2 wt%) CeO_2_	Sol–gel	150.37 mA h g^−1^ (3–4.5), capacity retention ∼76% (50 cycle)	

### 4.2 LiNiO_2_

**Fig. 6:**
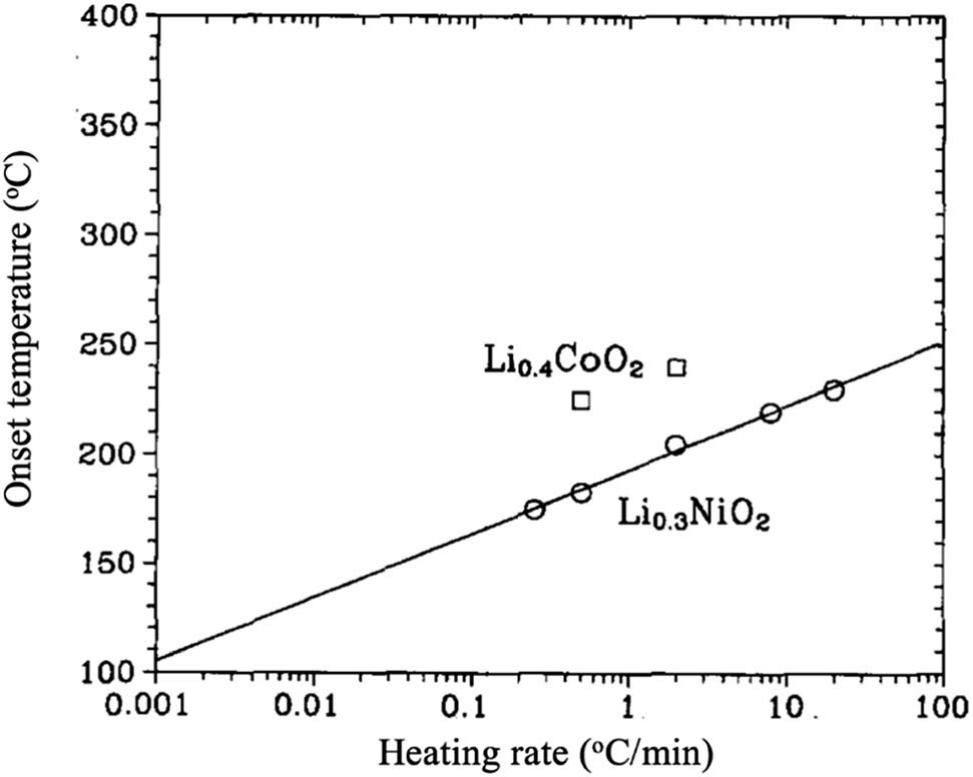
The onset temperature for oxygen release for Li_0.3_NiO_2_ and Li_0.4_CoO_2_ as a function of heating rate.^35^

LiNiO_2_ is considerably less expensive, shows an outstanding attainable capacity of 240 mA h g^−1^,^27^ and with less toxicity than LiCoO_2_. However, it is relatively difficult to synthesize stoichiometric LiNiO_2_ because of the preferable cation mixing between Li and Ni ions to form Li_1*z*_Ni_1+*z*_O_2_.^28,29^ The excess of Ni ions sitting in the lithium layers hinders the smooth motion of Li ions during cycling, resulting in poor performance. Moreover, the material goes through several structural transformations upon cycling. For the range 1 ≥ *x* ≥ 0.25, Li_*x*_NiO_2_ shows three single-phases; a rhombohedral phase (H1) when 1 > *x* > 0.75, a monoclinic phase when 0.75 ≥ *x* ≥ 0.45, a rhombohedral phase (H2) when 0.45 > *x* > 0.25, then a two-phase region (H2 + H3).^30,31^ In addition, Li_*x*_NiO_2_ is known for its high thermal instability as it decomposes and liberates oxygen at low temperatures compared to Li_*x*_CoO_2_ as shown in Fig. 6. The O_2_ evolution was found to be dependent on the degree of de-lithiation (*x*). Lee *et al.*^32^ synthesized and studied [Li_0.948_Ni_0.052_]_3a_[Li_0.003_Ni_0.997_]_3b_O_2_. When *x* ≥ 0.5, the material decomposes into layered LiNiO_2_ and spinel LiNi_2_O_4_ (*Fd*3*m*) in the range between 180 and 250 °C. In the composition range 0.5 > *x* ≥ 0.2, the material is completely transformed into spinel. Further increasing the temperature, the material turns into rock salt structure (*Fm*3*m*), with all transformations accompanied by oxygen evolution. It has been suggested that the exothermic reaction mechanism is driven by the phase transformations and oxygen evolution, which are originally due to the random mixing between Li and Ni ions.^33,34^ Recently, Yoon *et al.*^27^ successfully suppressed the H1 to H2 phase transition by preparing a compact geometry of LiNiO_2_ in which particles of 200 nm are packed tightly in a spherical particle of 10 microns. Such geometry minimized the contact between the electrolyte and the surface of the electrode resulting in better structure stability and capacity of 179 at 0.1 °C rate, 95% of the capacity was retained after 100 cycles when the cutoff voltage was 4.1 V. Similar to LiCoO_2_, better thermal, structural, and electrochemical stability is obtained by surface coating with ZrO_2_ or SiO_2_, Co and (Mn, Co) as shown in Table 3.

**Table 3:** LiNiO_2_ improved performance by coating Co and (Mn, Co) coating found to form a solid solution

Surface coating material	Technique	Electrochemical performance	Ref.
ZrO_2_	Sol–gel	190 mA h g^−1^ @ 36 mA g^−1^, voltage range (4.3–2.75 V)	36
		Capacity retention 98% (70 cycle)	
SiO_2_ (2 wt%)	Sol–gel	212 mA h g^−1^, @ 1C (3–4.5 V)	37
		Capacity retention (92.02% after 60 cycles)	
Co (content = 0.05)	Solid state synthesis	180.62 mA h g^−1^ @ 1C voltage range (2.75–4.2 V)	38
		Capacity retention 96.8% (50 cycle)	
Co and Mn	Solid state synthesis	181.3 mA g^−1^ voltage (3–4.35 V *vs.* Li/Li^+^)	39
		Capacity retention (91.1 after 50 cycles)	

### 4.3 LiCo_1−*x*_Ni_*x*_O_2_

LiCo_1−*x*_Ni_*x*_O_2_ was suggested as an alternative to LiNiO_2_ to merge the relatively better stability of LiCoO_2_ and the high capacity of LiNiO_2_. The LiCo_1−*x*_Ni_*x*_O_2_ solid solution can be formed in the whole *x* range. The lattice parameters and cell volume increase linearly while *c*/*a* decreases obeying Vegard's law when Ni ions are partially substituted for Co ions.^40^ The higher the Co ions content in LiCo_1−*x*_Ni_*x*_O_2_, the more stable the structure is. Co substitution suppresses the non-stoichiometry of Li_1−*z*_Ni_1+*z*_O_2_ from *z* = 0.04 to 0.01 when Co is increased from 0 to 0.2, and a pure stoichiometric 2D structure of LiCo_1−*x*_Ni_*x*_O_2_ was observed when Co content reached 0.3.^41^ The best performance of LiCo_1−*x*_Ni_*x*_O_2_ was obtained for 0.1 ≥ *x* ≥ 0.3.^42^ Differential scanning calorimetry for LiCo_1−*x*_Ni_*x*_O_2_ proved relatively higher safety and thermal stability than LiNiO_2_ at *x* = 0.1 ≥ *x* ≥ 0.3 at cutoff voltages of 4.2 and 4.3 V *vs.* Li/Li^+^.^43^ Upon increasing the Co content, the exothermic reaction shifts toward higher temperatures, and its enthalpy decreases. In addition, when the cell is charged to higher potential, the exothermic event takes place at a lower temperature in agreement with the fact that the lower the Li^+^ content, the lower the thermal stability of these oxide materials is. Recently, Xie *et al.*^44^ doped Li(Co,Ni)O_2_ with Mg ions to obtain highly structural, thermal, and electrochemical stable structure. Note that 80.1% of its initial capacity (214 mA h g^−1^) was maintained after 500 cycles compared to 56.3% without Mg ions. The Mg^2+^ prevented the Li^+^/Ni^2+^ site mixing, suppressed the migration of Ni ions to the Li layers, and showed a pillar effect in the position of the Li ions, which improved the thermal stability significantly even at such a high voltage as 4.7 V *vs.* Li^+^/Li.^45^Table 4 summarizes the thermal behavior of different transition metal oxides.

**Table 4:** Thermal behavior of different transition metal oxides

Composition	Decomposition temperature (°C)	Cutoff voltage (V)	Heat generated (J g^−1^)	Ref.
LiNiO_2_	181.2	4.3	1965	46
LiCoO_2_	212	4.5	2785	47
LiNi_0.9_Co_0.07_Mg_0.03_O_2_	243.7	4.3	543.2	45
LiNi_0.9_Co_0.07_Mg_0.03_O_2_	237.2	4.5	596.8	45
LiNi_0.9_Co_0.07_Mg_0.03_O_2_	211.6	4.7	667.5	45
Li[Ni_0.95_Co_0.025_ Mn_0.025_]O_2_	185.5	4.3	1609	46
LiNi_0.81_Co_0.19_O_2_	212.3	4.3	1095.2	48
LiNi_0.80_Co_0.19_Mg_0.01_O_2_	219.6	4.3	845.7	48
LiNi_0.78_Co_0_._19_Mg_0.01_Al_0.02_O_2_	221.9	4.3	394.6	48
Li[Ni_0.6_Co_0.2_Mn_0.2_]O_2_	201	4.3	1670	49
Li[Ni_0.9_Co_0.05_Mn_0.05_]O_2_	272	4.3	769	49
Li[Ni_1/3_Co_1/3_Mn_1/3_]O_2_	261.5	4.5	1998	47

### 4.4 LiMnO_2_

Manganese oxides are promising cathode materials due to their less toxicity and lower cost than cobalt and nickel oxides. Ion exchange is a successful method to synthesize layered LiMnO_2_ by exchanging Na in NaMnO_2_ with Li.^50^ Truly layered LiMnO_2_ with α-NaFeO_2_ like structure is thermodynamically unstable because of the Jahn–Teller effect that distorts the structure to less symmetry orthorhombic (*o*-LiMnO_2_) with the space group *Pmmn* or monoclinic (*m*-LiMnO_2_) with the space group *C*2/*m*.^51^ Monoclinic LiMnO_2_ is thermodynamically less stable than the spinel and orthorhombic lithium manganese oxides because of the antiferromagnetic interactions between Mn^3+^ distorting the MnO_6_ octahedra.^52^ This results in a transition from the initial monoclinic to hexagonal at the first charge then to spinel gradually upon cycling.^53^ This transformation takes place through the migration of 25% of Mn ions from the octahedral sites in the transition metal layer to the lithium layer, and the Li ions move to tetrahedral sites. Stabilization of the layered structure can be obtained by partially substituting Mn ions by antiferromagnetic elements such as Al or Cr.^54,55^ Many studies have been carried out on the structural and electrochemical properties of the Li–Ni–Mn–O and Li–Ni–Mn–Co–O systems as alternatives to the single oxides.

### 4.5 LiNi_0.5_Mn_0.5_O_2_

**Fig. 7:**
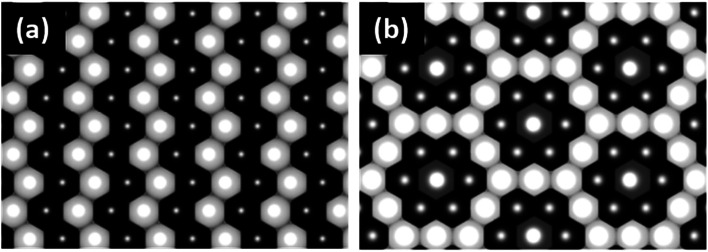
(a) TM layer ordering of the flower structure and (b) transition metal layer ordering of the zigzag structure.^59^

LiNi_0.5_Mn_0.5_O_2_ is a 4 V cathode material with a theoretical capacity of 280 mA h g^−1^. During charging, Ni^2+^ is oxidized to Ni^4+^ through two stages, while Mn^4+^ is electrochemically inactive and remains unchanged.^56,57^ The presence of the stable Mn^4+^ octahedral ions stabilizes the LiNi_0.5_Mn_0.5_O_2_ structure during Li^+^ removal, thus avoiding the Jahn–Teller distortion that is associated with the presence of Mn^3+^. There are different structural models that explain the composition of the transition metal layer in LiNi_0.5_Mn_0.5_O_2_; the most stable and lowest energy structures are shown in Fig. 7. The zigzag ordering of Ni^2+^ and Mn^4+^ is preferred if there is no Li^+^/Ni^2+^ mixing, while the flower order is preferred if there is Li^+^/Ni^2+^ mixing. The flower structure is ordered in such a way that the Li^+^ is located in the center of an Mn^4+^ hexagon surrounded by Ni^2+^.^58,59^ LiNi_0.5_Mn_0.5_O_2_ is indexed in the *R*3̄*m* space group, with around 8 to 10% anti-cite mixing between Li and Ni usually takes place.^60^ Li^+^ prefers to occupy positions close to Mn^4+^ ions than Ni^2+^ in the transition metal layer.^58^ Therefore, Li, Ni, and Mn ions are not randomly distributed in the transition metal layer. Single crystal selected area electron diffraction patterns of pristine LiNi_0.5_Mn_0.5_O_2_ reveal the presence of superlattice reflections indicating the long-range ordering of Li-rich and Li-deficient sites in the transition metal layer. The superlattice reflections are indexed in 
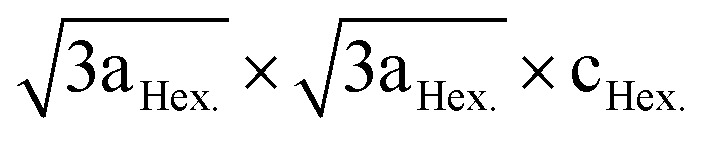
 and correspond to a trigonal structure with the space group *P*3_1_12.^61,62^ Investigation of the local structure upon charging suggested the disappearance of Li^+^ located in the transition metal layer very early during the first charge.^63^ Li^+^ migrates to the tetrahedral sites that share faces with vacant octahedral sites in the transition metal layer. The presence of Li in the tetrahedral sites reflects negatively on the electrochemical performance of the material. Because Li_tet_^+^ is very energetically stable and requires high potential to be removed, which decreased the capacity obtained in the practical voltage range.^64^ Regarding the Ni ions in the Li layer, by charging above 4.6 V, 75% of Ni_Li_ ions migrate to vacant Li positions in the transition metal (TM) layer.^65^ The migration of Ni ions to the TM layer is partly a reversible process and reflects on the weaken or disappearance of the 
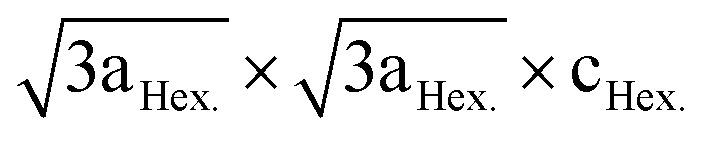
 c supercell. Simply, in other words, the Ni^2+^/Li^+^ mixing was decreased by cycling compared to pristine LiNi_0.5_Mn_0.5_O_2_.

### 4.6 LiNi_*x*_Co_*y*_Mn_1−*x*−*y*_O_2_

The mixed transition metal oxides family LiNi_*x*_Co_1−2*x*_Mn_*x*_O_2_ (ref. 66) was proposed to avoid the shortcoming of each individual oxide such as low stability, safety, and specific capacity. The cation mixing in this compound is lower than at LiNi_0.5_Mn_0.5_O_2_, and it increases by increasing the Ni content. LiNi_1/3_Co_1/3_Mn_1/3_O_2_ was found to be promising among the series of materials in the composition range 0 ≤ *x* ≤ ½. LiNi_1/3_Co_1/3_Mn_1/3_O_2_ showed a specific capacity of 200 mA h g^−1^ when charged up to 4.6 V *vs.* Li/Li^+^. It crystallizes in the typical layered structure of α-NaFeO_2_ with *R*3̄*m* space group with the electronic charge of Co, Ni, and Mn being +3, +2, and +4, respectively. Several authors debated its electronic structure upon the de-intercalation of lithium. Tsai *et al.* and Yoon *et al.* reported that its electrochemical performance is attributed to the redox reactions Ni^2+^/Ni^3+^ at the surface and Ni^3+^/Ni^4+^ in bulk, while the oxidation states of Mn^4+^ and Co^3+^ remain unchanged.^67,68^ In addition, K-edge XAS of the oxygen ions shows that it plays an essential role in the charge compensation in its site during Li^+^ de-intercalation. On the other hand, Kim *et al.*^69^ reported that the charge compensation during charging is due to the redox Ni^2+^/Ni^4+^ and Co^3+^/Co^4+^ while Mn^4+^ is stable.

### 4.7 Li-rich cathode materials

#### 4.7.1 Li_2_MnO_3_

Li_2_MnO_3_ is one of the *x*Li_2_MnO_3_·(1 − *x*)LiMO_2_ family of materials when *x* = 0. It possesses layered structure Li_3a_[Li_1/3_Mn_2/3_]_3b_O_2_ with *C*2/*m* space group. In Li_2_MnO_3_, the Mn^4+^ is electrochemically inactive because it cannot be oxidized to a higher oxidation state. Surprisingly, Li^+^ could be inserted and extracted from Li_2_MnO_3_ when activated at 4.5 V.^70–72^ This material has promoted many debates on the mechanism of the electrochemical behavior and Li^+^ extraction. During the first charge, the Li^+^ extraction was found to be accompanied by oxygen evolution, leading to the removal of Li_2_O, resulting in electrochemical activation of Li_2_MnO_3_.^73^ Robertson *et al.* suggested that there is a proton exchange with Li^+^ to form Li_2−*x*_H_*x*_MnO_3_,^74^ which can take place after the production of hydrogen by electrolyte oxidation. The presence of H in the oxide layers forming O–H–O bond leads to a change to a more stable stacking sequence from O3(ABCABC) to P3(ABBCCA). Another proposed mechanism based on DFT calculations shows that the electrochemical behavior is attributed to the charge compensation by the oxidation of anion “oxygen”.^75^ However, the thermodynamically unstable localized holes at the beginning of the oxidation result in the evolution of oxygen. This study showed that the structural transformation to spinel is driven by the oxygen dimerization, which facilitates the Mn ions migration onto octahedral sites in the vacated lithium layers. Rana *et al.* suggested that both oxygen oxidation and proton exchange are responsible for the electrochemical behavior of Li_2_MnO_3_.^76^ Oxygen and Li^+^ are removed from the material, leaving layered Mn_2_O and the structure change from O3 to P3 due to the proton–Li^+^ exchange. This structural change is reversible, and it turns to O3 during the following discharge, but there is an irreversible oxygen loss due to the material activation during the first cycle. The repeated Li^+^–H^+^ exchange, which leads to the change from O3 to P3 and the reverse upon cycling, was found to be responsible for the poor electrochemical performance of Li_2_MnO_3._ The repeated shearing of oxygen layers results in permeant damage of the structure upon cycling.

#### 4.7.2 *x*Li_2_MnO_3_·(1 − *x*)LiMO_2_

**Fig. 8:**
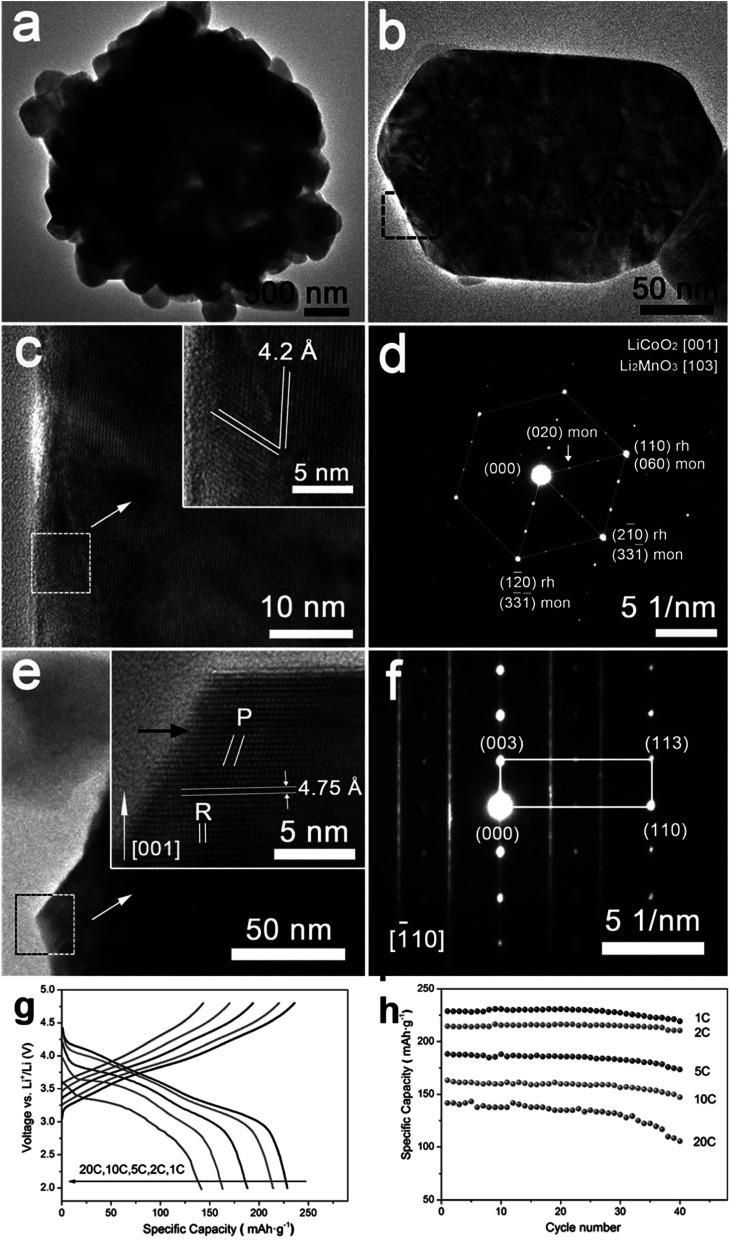
(a–f) Hierarchical Li_1.2_Ni_0.2_Mn_0.6_O_2_ nanoplates with exposed {010} planes as high-performance cathode material for lithium-ion batteries, (g) discharge curves of half cells based on the Li_1.2_Ni_0.2_Mn_0.6_O_2_ hierarchical structure nanoplates at 1C, 2C, 5C, 10C, and 20C rates after charging at C/10 rate to 4.8 V, and (h) the rate capability at 1C, 2C, 5C, 10C, and 20C rates.^88^

*x*Li_2_MnO_3_·(1 − *x*)LiMO_2_ (M = Ni, Co, Cr) are attractive materials due to their high-energy density and low cost. Li[Li_1/3−2*x*/3_Ni_*x*_Mn_2/3−*x*/3_]O_2_ is a layered oxide composed of two phases: monoclinic *x*Li_2_MnO_3_ and hexagonal (1 − *x*)LiMO_2_ integrated into a single nanoparticle.^77,78^ In the range 0 ≤ *x* ≤ 1/3, the Li and transition metals are ordered in the transition metal layer on a 
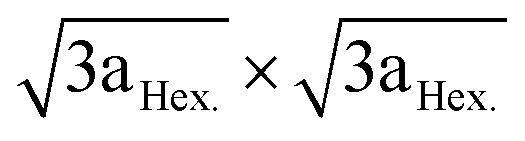
 super structure.^79^ It exhibits high voltage during charging (>4.5 V), high specific capacity (>250 mA h g^−1^), and high energy density.^70,80,81^ The electrochemical behavior of Li-rich, layered oxides is divided into two stages. The first is before 4.4 V when the Li^+^ is extracted from LiMO_2_. The second starts at 4.5 V when the Li_2_O is extracted to activate Li_2_MnO_3_. The Li-rich, layered oxides suffer from some drawbacks such as an irreversible capacity loss due to the irreversible extraction of Li_2_O and the reaction with electrolyte at high potential, poor rate capability due to high charge transfer resistance and a voltage fade upon cycling. The origin of the voltage fade was suggested to be the transition from layered to spinel, which is accompanied by an irreversible transition metals migration to the Li-ions layers.^82–85^ Despite the fact that the nanomaterials with the high surface area are known for their undesired side reactions with the electrolyte, reduction of particle size of Li-rich materials to the nanoscale shows a better rate capability and a decrease in the diffusion path of Li ions.^86,87^ Moreover, decreasing the interface area between the electrode and the electrolyte by controlling the morphology of the nanomaterials could suppress the erosion from the electrolyte and improve the cycling performance. Taking Li_1.2_Ni_0.2_Mn_0.6_O_2_ as an example, hierarchical nanoplates structure shows outstanding rate capability as shown in Fig. 8, with specific capacity of ∼231, 216.5, 188, 163 and 142 mA h g^−1^ at as high rates as 1C, 2C, 5C, 10C, and 20C.^87^ Furthermore, it shows high capacity retention of 95.5% and 86.6% after 60 cycles for 1C and 2C rates, respectively. The classical voltage decay of Li-rich material was still observed due to the transition to spinel. An expected significant decrease in capacity retention to 68% occurred when the hierarchical structure was damaged by ultrasonic treatment.

## 5 Spinel positive electrode materials

### 5.1 LiMn_2_O_4_

Spinel LiMn_2_O_4_ is one of the most attractive energy storage materials due to its low cost and reversible and fast intercalation and de-intercalation of Li ions.^89–91^ The LiMn_2_O_4_ spinel structure (space-group: *Fd*3*m*), consists of oxygen ions close-packed array occupy the 32e position, Mn ions occupy the octahedral site 16d, and Li^+^ occupies the tetrahedral 8a site. In Li_*x*_Mn_2_O_4_ (0 < *x* ≤ 2), Mn_2_O_4_ provides a 3-dimensional host network for Li^+^. When the voltage is ∼4 V, Li ions are hosted in the 8a site in the range 0 < *x* ≤ 1. However, at 3 V, the inserted Li^+^ ions occupy the octahedral 16c sites, which share faces with the 8a tetrahedral. The Li-ions at the 8a sites move to the empty 16c sites, resulting in a first-order phase transition to Li_2_Mn_2_O_4_. In addition, when Mn^3+^ concentration increases, Jahn–Teller distortion occurs to decrease the crystal symmetry from cubic to tetragonal Li_2_Mn_2_O_4_. Therefore, it is believed that the electrochemical performance of spinel LiMn_2_O_4_ is better at 4 V than 3 V. However, it is still possible to observe the presence of Li_2_Mn_2_O_4_ on the surface of LiMn_2_O_4_ in a 4 V cell at the end of the discharge process as an over-discharge product.^92^ Then, MnO could be dissolved from Li_2_Mn_2_O_4_, resulting in the formation of Li_2_MnO_3_ on the surface of LiMn_2_O_4_.^93^ MnO dissolution is attributed to the formation of HF acid from the electrolyte.

1 Li_2_Mn_2_O_4_ → Li_2_MnO_3_ + MnO

Generally, LiMn_2_O_4_ suffers from a capacity fading problem, especially at a temperature of 55 °C due to the phase transitions discussed earlier as well as the chemical instability of the spinel LiMn_2_O_4_ with the electrolyte, resulting in the dissolution of Mn ions.^94^ The improvement of the electrode and electrolyte interface region by providing a protective coating on the material surface to decrease the direct contact with the electrolyte is one of the most extensively studied methods to avoid the previously mentioned problems. Electrochemically active materials coatings, such as LiNi_0.5_Mn_1.5_O_2_, LiCoO_2_, Li_4_Ti_5_O_12_, and metal oxide coating by B_2_O_3_, ZnO, or TiO_2_, show improved performance of the material at room temperature and 55 °C.^95–99^ Doping with Al, Co, Cr, or Ni improved the cyclability but at the expense of the initial capacity.^100,101^ Furthermore, the anionic substitution by F, along with the cationic substitution, show improved performance.^102,103^

### 5.2 LiNi_0.5_Mn_1.5_O_4_

The substitution of 25% of Mn for Ni ions in the spinel LiMn_2_O_4_ results in one of the most promising materials for high energy applications (LiNi_0.5_Mn_1.5_O_4_) because it shows one dominant voltage plateau at 4.7 V, while another doped LiMn_2_O_4_ show two plateaus between 4 and 5 V. Electrochemical performance of spinel LiNi_0.5_Mn_1.5_O_2_ is attributed to the Ni^2+^/Ni^4+^ redox at 4.7 V with Mn^4+^ remains unchanged. LiNi_0.5_Mn_1.5_O_2_ orders in two different space groups *Fd*3*m* or *P*4_3_32. The spinel type that exhibits *Fd*3*m* space group is a disordered phase that has a face centered cubic structure in which Li ions occupy the 8a sites, the transition metals Ni and Mn ions randomly occupy the 16d sites, and the ccp oxygen array occupies the 32e sites. The spinel type that exhibits *P*4_3_32 space group is an ordered phase that has a primitive cubic structure in which the Li ions occupy the 8c positions, Mn and Ni are regularly ordered in the 12d and 4a sites, respectively, while the oxygen ions occupy both the 24e and 8c positions.^104^ The disordered structure exhibits better performance than the ordered spinel structure.^105^ Despite its voltage is considered as one of the highest among other available materials, this voltage is outside the stability window of the electrolyte, which leads to the formation of SEI that affects the kinetics of the electrochemical insertion and de-insertion of Li ions. The stability of the SEI can be improved by surface modification *via* coating.^106^ In addition, coating plays a beneficial role in suppressing the dissolution of Mn ions from Li Ni_0.5_Mn_1.5_O_4_.

## 6 Polyanionic positive electrode materials

### 6.1 Phosphates-based cathode materials

**Fig. 9:**
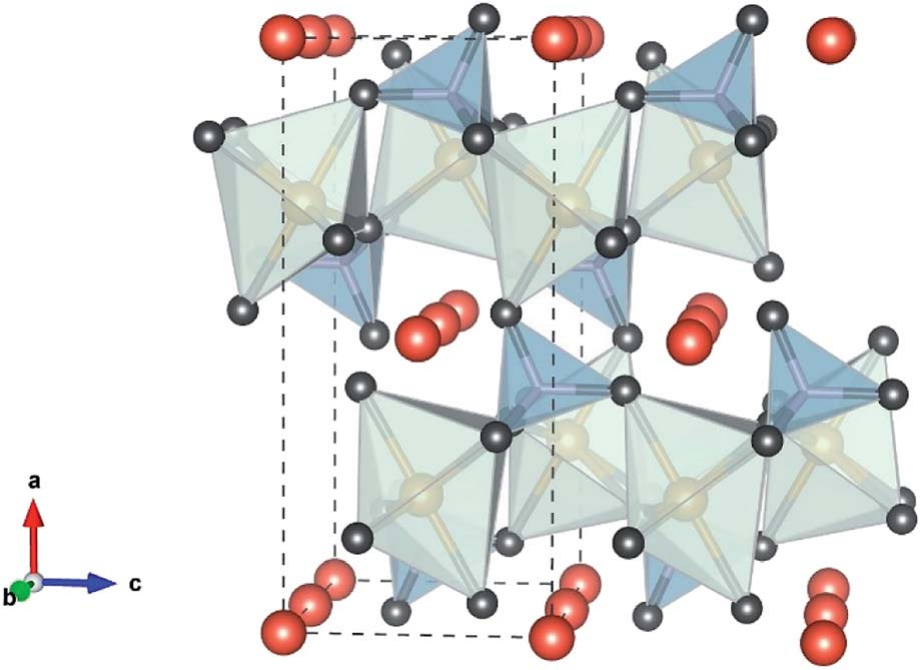
Polyhedral representation of the olivine structure. FeO_6_ (light green), PO_4_ (gray), and the Li^+^ (orange) “by VESTA”.

Polyanionic olivine LiMPO_4_ (M = Fe, Mn, Co or Ni) are very attractive as cathode materials for LIBs. LiMPO_4_ has an olivine structure, Fig. 9, and crystallizes in the orthorhombic *Pnma* space group in which the oxygen ions form a hexagonal close-packed framework, Li occupies the octahedral M1 site (4a) forming a tunnel along the direction [010], the transition metal (Fe, Ni, Mn, or Co) ion is located in the M2 site (4c) forming a zigzag chain of corner shared octahedra linked to the phosphate tetrahedra. The MO_6_ octahedral and PO_4_ tetrahedral share their edges. The strong P–O covalent bond provides high thermodynamic stability and prevents oxygen evolution at high temperatures compared to LiCoO_2_ and LiNiO_2_. This strong PO_4_^3−^ shows lower redox energy of 3d orbital of the metal than the Fermi level. Therefore, the P–O covalent bond is responsible for the lower potential in olivine than oxide materials. The cell parameters of LiMPO_4_ (M = Fe, Mn, Co, or Ni) are given in Table 5.

**Table 5:** Cell parameters of LiMPO_4_ (M = Fe, Mn, Co or Ni)

LiMPO_4_	*a*	*b*	*c*	Ref.
LiFePO_4_	10.3377(5)	6.0112(2)	4.6950(2)	107
LiMnPO_4_	10.4472(5)	6.11049	4.7459(2)	108
LiCoPO_4_	10.20	5.92	4.70	109
LiNiPO_4_	10.060	5.776	4.683	110

#### 6.1.1 LiFePO_4_

LiFePO_4_ is the most attractive commercialized cathode material because of its desirable safety features, high theoretical capacity (170 mA h g^−1^) at moderate current densities, stable Fe^3+^/Fe^4+^ redox potential of 3.5 V *vs.* Li^+^/Li, flat voltage plateau, thermal stability even at high temperatures, stable electrochemical and chemical properties, excellent cycling performance, low cost, and abundance of iron. During the insertion and removal of Li^+^, it was observed that there are two coexisting phases, LiFePO_4_ and FePO_4_. Both phases have the same space group (*Pnma*). Padhi *et al.* suggested that the insertion and extraction of Li^+^ is associated with the motion of the two-phase interface.^111^ They suggested the shrinkage core model in which the Li ions move from the surface of a particle inward through the phase boundary. Upon lithiation, the surface area of the interface decreases until it reaches a critical surface area when the rate of Li insertion is not able to sustain the applied current. This makes LiFePO_4_ a suitable material for low power applications as it influences the performance of the material in terms of capacity and rate capability at high current density. Srinivasan *et al.* assumed a juxtaposition of the LiFePO_4_ and FePO_4_ phases and suggested a shrinking core model in which the shell of one phase covers the core of the other phase by considering that the Li-ion diffusion drives the phase boundary motion.^112^

**Fig. 10:**
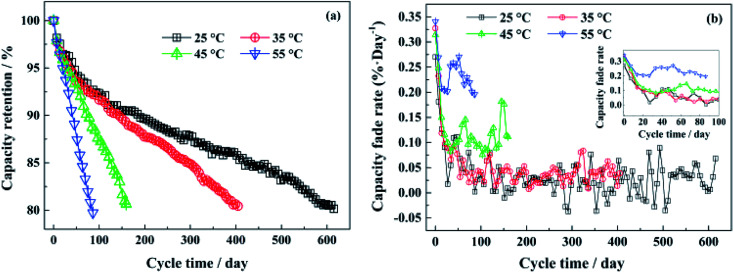
(a) The capacity retentions of the LiFePO_4_/graphite full cells at different temperatures with continuous cycling days, (b) the capacity fade rate at each test temperature during cycling.^113^

The battery cycling and calendar age influence the cycle life, capacity, and power capability of LIB. The factors that influence the performance can be a function of either the operation conditions, such as temperature or working potential window, or the type of the cathode material. For example, Sun *et al.* showed that the high temperature to have a tremendous negative impact on the performance of the LiFePO_4_ battery.^113^ The battery lifetime at room temperature is about seven times higher than that at 55 °C as presented in Fig. 10. The reason for capacity loss at room temperature is assigned to the irreversible loss of Li by the formation of a solid electrolyte interface (SEI) layer. Upon increasing the ambient temperature, the interplanar spacing between the crystallographic planes of the structure becomes larger, and the surface layer (SEL) becomes thicker, which is a sign of degradation. Also, the SEM images of the surface of the aged cathodes at temperatures higher than 45 °C showed a rounded-shaped particle rich in fluorine with the particle size increased from 0.7–0.8 μm to 1.3–1.5 μm with increasing the temperature, which is accompanied by the accelerated decomposition of the electrolyte.

**Fig. 11:**
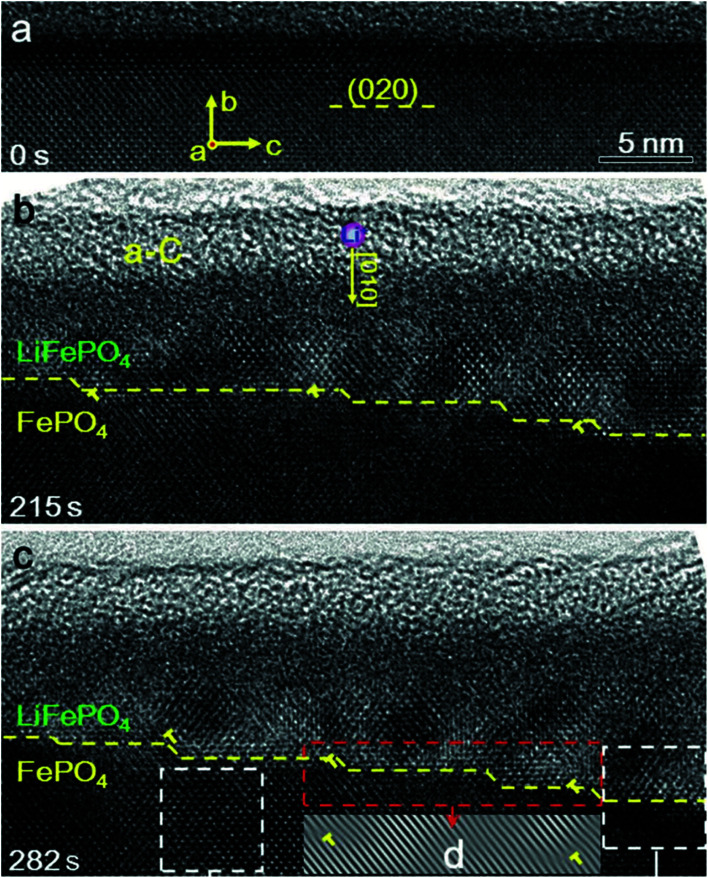
Phase boundary between FePO_4_ and LiFePO_4_ and its migration along the [010] direction during lithiation. (a) A HRTEM image of the pristine FePO_4_ (b) after 215 second from applying the voltage, a step-like phase boundary was formed between FePO_4_ and LiFePO_4_, as pointed out by the yellow dashed line. (c) At 282 seconds, the thickness of the LiFePO_4_ layer increased as the step-like phase boundary propagating along the [010] direction.^114^

There is a structural instability that usually occurs during the intercalation and de-intercalation process of Li, which results in fast capacity fading upon cycling. A study used *in situ* TEM and HRTEM analyses showed that the lithiation and de-lithiation in LiFePO_4_ occur by two phase reactions (LiFePO_4_ and FePO_4_).^114^ Upon applying potential to FePO_4_, Li ions inserted into the structure to form a layer of LiFePO_4_ that gets thicker with time, which was evident *via* the migration of the step shape phase boundary along the *b* axis or [010] direction as shown in Fig. 11. This study proves that there is a lattice mismatch that induced dislocation at the phase boundary, where the transformation between the two phases produced an array of dislocations in the FePO_4_ side. Also, the orientation of LiFePO_4_ changed slightly compared to FePO_4_ as a result of the elastic deformation that can accommodate the transformation strain. These dislocations accumulate during the cycling and can cause further defects/cracks that cause degradation. Moreover, the ageing of C_6_/LiFePO_4_ (LFP) batteries was studied theoretically by the electron-tunneling-based model for lithium immobilization.^115^ The SEI formation model presumes the existence of a porous outer and dense inner SEI layers. Electron tunneling through the inner SEI layer is considered to be the rate-determined step in the SEI formation process and its initial thickness after activation process controls the degradation rate. The outer SEI layer grows much faster than the inner layer. During cycling, cracks formation takes place, exposing the new surface of graphite to the electrolyte directly resulting in the formation of SEI, hence loss in capacity. The formed SEI was found to be dependent on the number of cycles.

**Fig. 12:**
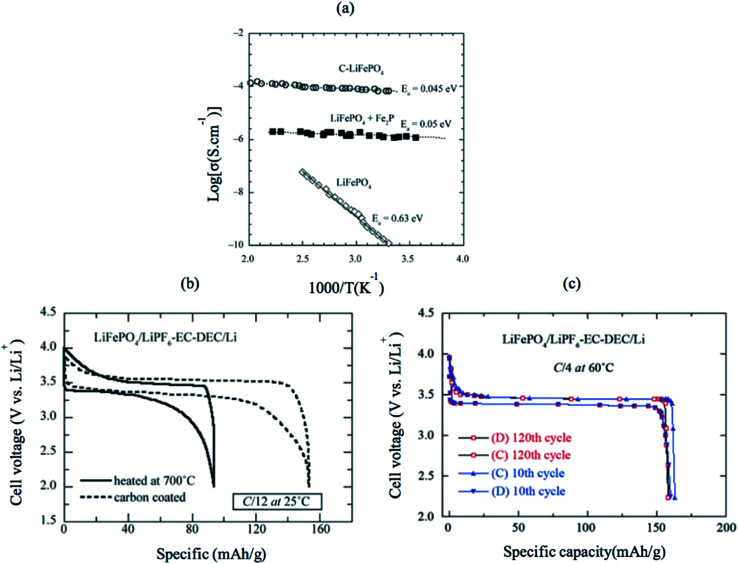
(a) Electronic conductivity of pure LiFePO_4_, Fe_2_P-containing sample and C/LiFePO_4_. (b) The electrochemical profiles at C/12 of non-coated and heated at 700 °C LiFePO_4_//Li cells (c) electrochemical performance of the C–LiFePO_4_//Li cell operating at 60 °C. Charge–discharge cycling was conducted at the C/4 rate (about 35 mA g^−1^) in the voltage range 2.2–4.0 V *vs.* Li^0^/Li^+^.^123^

LiFePO_4_ has a low intrinsic electronic conductivity of 10^−9^ S cm^−1^ and low ionic conductivity. Several attempts were carried out to increase both its ionic and electronic conductivity and, consequently, its electrochemical performance. First, conductive surface coating by carbon was found to improve the electronic conductivity due to the improved electric contact between the electrode particles, as shown in Fig. 12a.^116^ Several groups tailored the C/LiFePO_4_ composite electrode and obtained specific capacity close to the theoretical limits of LiFePO_4_ (170 mA h g ^−1^). Furthermore, C-coating prevents particle growth during heat treatment resulting in the synthesis of smaller particle size and higher surface area LiFePO_4_.^117^Fig. 12b shows a comparison between the electrochemical profile of the bare and C-coated LiFePO_4_ at C/12 rate; both have the same particle size (40 nm). The C-coated sample showed high capacity that is close to the theoretical limit, while bare LiFePO_4_ showed only 55% of the theoretical capacity. Even at higher rate of C/4, C/LiFePO_4_ shows excellent Li ions extraction kinetics and outstanding ability to maintain 100% of its initial capacity (160 mA h g^−1^) after 120 cycles. However, the addition of additives such as C would increase the conductivity, but it decreases the energy density of the cathode because it is counted as electrochemical inactive material. Carbon-free LiFePO_4_ small particles with narrow size distribution can shorten the ionic and electronic diffusion length and improve the conductivity of the material.^118,119^ Delacourzt *et al.* prepared 100–200 nm LiFePO_4_, which showed a high reversible specific capacity of 145 mA h g^−1^ at C/2. Third, the electronic conductivity and Li ion diffusion in LiFePO_4_ structure would be improved by doping at the anionic and/or cationic sites. Chung *et al.* reported that multivalent ions-doped LiFePO_4_ showed outstanding electronic conductivity of eight orders of magnitude higher than pure LiFePO_4_.^120^ However, the reason for the improved conductivity is questioned since Herle *et al.*^121^ showed that the iron salt precursor in Chung's experiment was the source of C, which can reduce Fe and P to Fe_2_P and/or Fe_3_P (conductivity of LiFePO_4_ + Fe_2_P is shown in Fig. 12a). Therefore, the metal-rich phosphides are believed to be responsible for the improved conductivity. A combination of C and Zn^4+^ doping showed a synergetic effect that facilitated electron transport.^122^

#### 6.1.2 LiMnPO_4_

**Fig. 13:**
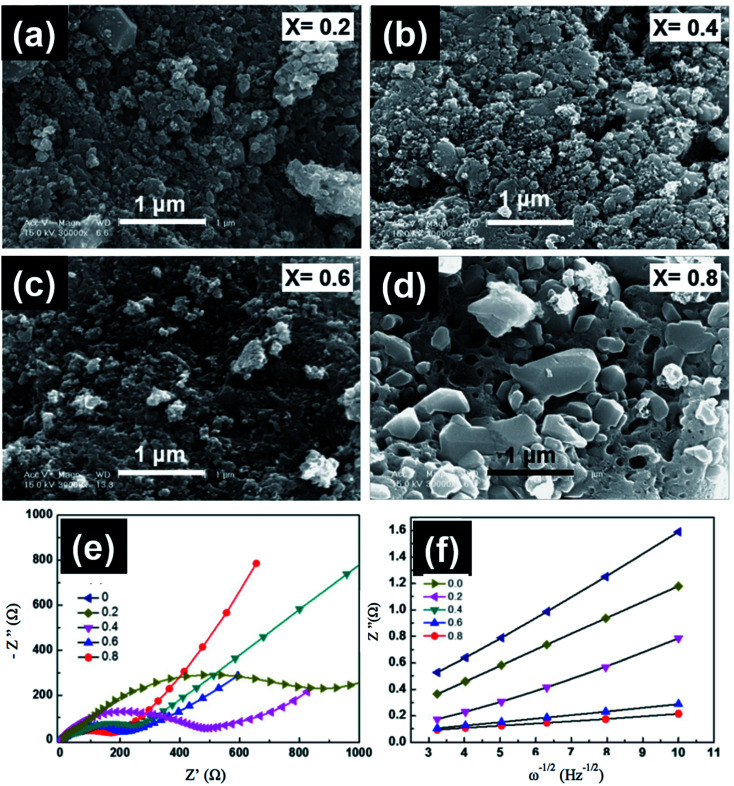
(a–d) FESEM images, (e) impedance spectra, and (f) the relationship between *Z*′′ and the square root of frequency (*ω*^−1/2^) in the low-frequency region for LiMn_1−*x*_Fe_*x*_PO_4_ at different compositions.^131^

The low energy density of LiFePO_4_ restricts their use in the automotive industry. The study and development of other members from the olivine family with higher discharge voltage and energy density, such as LiMnPO_4_, are critical. LiMnPO_4_ showed higher potential than LiFePO_4_ (4.1 V *vs.* Li^0^/Li^+^) and a high energy density of ∼700 W h kg^−1^.^124^ However, cyclic stability and rate capability of LiMnPO_4_ are hindered by the poor kinetics and low ionic and electronic conductivity. Furthermore, the presence of Mn^3+^ ions in an unstable position in the lattice leads to cell distortion, which is controlled by the Jahn–Teller effect.^125^ Similar to LiFePO_4_, several strategies were used to improve the conductivity of LiMnPO_4_, such as the synthesis of smaller particle size, carbon coating, and cation substitution by Fe, Mg, or Zn.^126–128^ The solid solution of LiMnPO_4_ and LiFePO_4_ operates in the voltage range between 3.4 and 4 V *vs.* (Li/Li^+^), which is not very high to prevent the electrolyte decomposition and at the same time offers high energy density with respect to LiFePO_4_ and better rate capability than LiMnPO_4_.^129^ A common defect of anti-site mixing between Fe/Mn and Li ions in the mixed LMFP is dependent on the synthesis method, and it affects the Li ions motion along the 010 channel upon cycling. Sun *et al.*^130^ studied the effect of Fe substitution on the conductivity and Mn ions dissolution. They prepared nanostructured LiFe_*x*_Mn_1−*x*_PO_4_ (*x* = 0 and 0.15) by spray pyrolysis and suggested that the performance was improved because the conductivity increased, and Mn dissolution decreased from 103.7 to 87.2 ppm. Seo *et al.*^131^ studied the charge transfer resistance (*R*_ct_) and the electrochemical performance of LiFe_*x*_Mn_1−*x*_PO_4_ at *x* = 0, 0.2, 0.4, 0.6, 0.8. The best electrochemical performance was observed when *x* = 0.8 and 0.6, which corresponded to the highest discharge capacity of almost 154 and 149 mA h g^−1^ and retained 100% and 101% of its initial capacity after 50 cycles, respectively. The impedance patterns of all compositions showed a single semicircle in the high frequency region as shown in Fig. 13. The semicircle at high frequencies is due to the combination between double layer capacitance and *R*_ct_. The *R*_ct_ decreased by increasing the Fe content and reached its lowest value of 170 Ω when *x* = 0.8. The Warburg impedance at low frequency is related to the diffusion of Li ions. The Li ions diffusion increased with decreasing the slope of the line and reached its highest value of 7.33 × 10^−14^ when *x* = 0.8. Dai *et al.* reported that the co-substitution of Mn by Fe and Mg could significantly improve the cycling performance, where the optimal composition was found to be LiMn_0.8_Fe_0.19_Mg_0.01_PO_4_.^132^ Moreover, they studied the effect of C content on the electrochemical performance of this composition. The material with 10.5 wt% carbon showed the best rate capability and capacity of 143.1 mA h g^−1^, 130 mA h g^−1^, and 125.7 mA h g^−1^ at 1C, 5C, and 10C rates, respectively. Lower C content of 4.1 wt% showed a slight decrease in the rate capability. However, further decrease or increase in the C content to 2 wt% or 27.4 wt% resulted in fast capacity fade at high C rate.^133^ This better performance is explained by the lowest charge transfer resistance of ∼27 Ω at 10 wt% C electrodes.

#### 6.1.3 LiCoPO_4_ and LiNiPO_4_

**Fig. 14:**
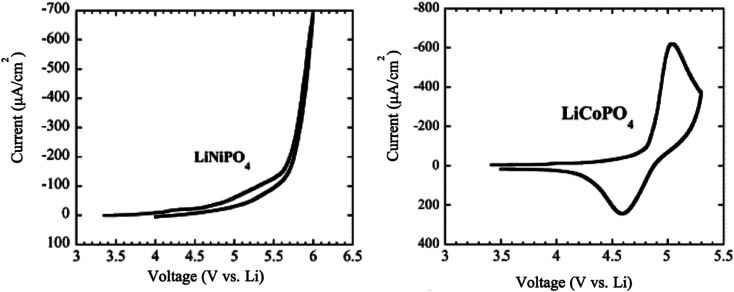
Cyclic voltammetry of LiNiPO_4_ and LiCoPO_4_ at a scan rate of 0.2 mV s^−1^.^135^

The LiCoPO_4_ and LiNiPO_4_ materials exhibit a high potential of 4.8 V and 5.2–5.4 V, respectively.^134^ It is challenging to extract lithium from LiNiPO_4_ within the stable voltage window of the available electrolytes. Moreover, both compounds showed much lower electronic conductivity than LFP and LMP. The cyclic voltammetry of LiCoPO_4_ showed that there is a reduction peak at 4.6 V and an oxidation peak at 5.1 V, while there are no significant reduction peaks for LiNiPO_4_ in the range between 3.5 to 6 V *vs.* Li/Li^+^ as shown in Fig. 14.^135^

### 6.2 Silicate-based cathode materials

The replacement of phosphate by silicate was a crucial step in the search for new high energy cathode materials. Orthosilicate cathode materials (Li_2_MSiO_4_, M = Fe, Mn) are of particular interest due to their superior electrochemical performance, safety, raw material abundance, and low cost. Li_2_MSiO_4_ has a high theoretical specific capacity (∼333 mA h g^−1^) if the two Li ions are fully extracted per formula unit. Several temperatures and pressure-dependent polymorphs of Li_2_MSiO_4_ exist.^136^ The framework consists of distorted hexagonal close backed oxygen array, and all cations occupy tetrahedral sites. The different possible cations ordering produce different polymorphs. These polymorphs can be divided into low or high temperature, which corresponds to β or γ Li_3_PO_4_ like structures, respectively. Each type could be explained in several space groups such as orthorhombic β_I_ (*Pbn*2_1_, *Pna*2_1_) and β_II_ (*Pmn*2_1_), monoclinic γ_s_/γ_0_ (*P*2_1_/*n*), and orthorhombic γ_II_ (*Pmna*, *Pmnb*).

#### 6.2.1 Li_2_FeSiO_4_

**Fig. 15:**
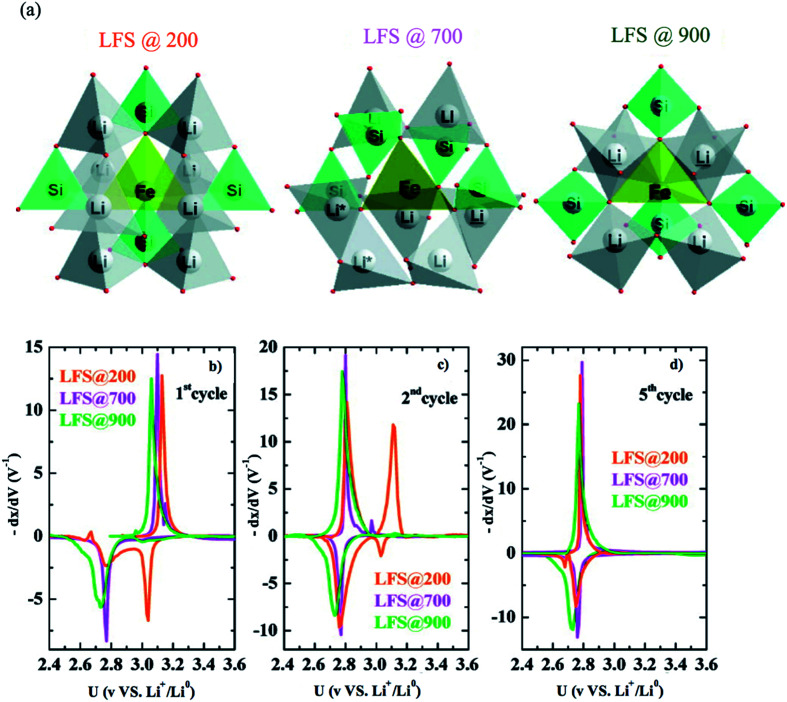
(a) The three polymorphs of Li_2_FeSiO_4_, derivative plots obtained from PITT measurements in the (b) first, (c) second, and (d) fifth cycles for all three polymorphs.^141^

Li_2_FeSiO_4_ exhibits a specific capacity of 166 mA h g^−1^ since only one Li^+^ can be extracted due to the redox couple Fe^3+^/Fe^2+^ at 3.1 V *vs.* Li^+^/Li. The lower electronegativity of Si compared to P results in a lowering of the Fe^3+^/Fe^2+^ redox couple in Li_2_FeSiO_4_ than in LiFePO_4_. The other redox couple Fe^4+^/Fe^3+^ takes place at a high voltage of 4.85, which is outside the stability window of the electrolyte; therefore, it is not reached. Li_2_FeSiO_4_ exhibits complex structural defects that make it difficult to analyze its structure. Nytén *et al.*^137^ reported Li_2_FeSiO_4_ in the orthorhombic system with *Pmn*2_1_ space group and explained two additional peaks in its diffractogram as an unidentifiable impurity. Its potential shifts irreversibly from 3.1 V to 2.8 V in the second cycle. This voltage drop was suggested to be due to site mixing between Li ions and Fe ions since the Li : Fe ratio in the 4b site changed from 96 : 4 to 40 : 60 during the first cycle.^138^ Nishimura *et al.*^139^ reported Li_2_FeSiO_4_ in the monoclinic system and *P*2_1_ space group. Sirisopanaporn *et al.*^140^ synthesized a polymorph, which is an iso-structural with Li_2_CdSiO_4_ and is described by the *Pmnb* space group. Sirisopanaporn *et al.*^141^ compared the electrochemical behavior of Li_2_FeSiO_4_ polymorphs, which were explained in the *Pmn*2_1_, *P*2_1_/*n*, and *Pmnb* space groups by synthesis under hydrothermal conditions at 200, 700, and 900 °C, respectively. The structural difference of these polymorphs is shown in Fig. 15a. Each polymorph showed different first oxidation potential. The *Pmnb* polymorph exhibited the lowest potential (2.9 V *vs.* Li^+^/Li^0^), *P*2_1_/*n* showed an intermediate potential of 2.97 *vs.* Li^+^/Li^0^, while *Pmn*2_1_ showed the highest oxidation potential (3.1 V *vs.* Li^+^/Li^0^). Derivative plots obtained from PITT of the three polymorphs showed that they all experience the voltage fade from 3 to 2.8 V upon cycling due to the transformation to Li-poor phase as shown in Fig. 15b–d. More precisely, it showed that the high temperature *Pmnb* and *P*2_1_/*n*, which have high degree of disorder, transformed faster than the low temperature *Pmn*2_1_. The first discharge reduction potential of *Pmnb* and *P*2_1_/*n* was ∼2.76 V *vs.* Li^+^/Li^0^, while the discharge of *Pmn*2_1_ showed two reduction peaks at ∼2.76 and ∼3.04 V *vs.* Li^+^/Li^0^ indicating the presence of initial Li_2_FeSiO_4_. The high-temperature *Pmnb* and *P*2_1_/*n* experienced a phase transformation at the first cycle faster than *Pmn*2_1_, which is fully transformed at the fifth discharge.

#### 6.2.2 Li_2_MnSiO_4_

**Fig. 16:**
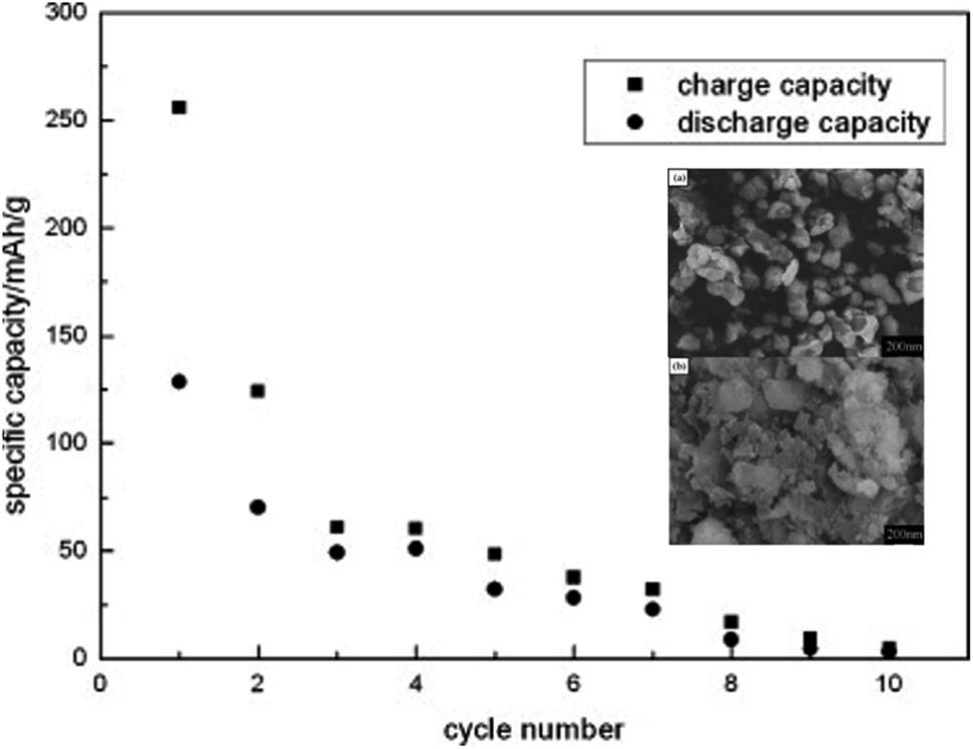
Cyclic performance of Li_2_MnSiO_4_ synthesized by solid state method tested at C/16 rate to a cut-off voltage between 1.5 and 4.8 V.^152^ The inset shows FESEM micrographs of (a) Li_2_MnSiO_4_ and (b) Li_2_MnSiO_4_/C.

**Fig. 17:**
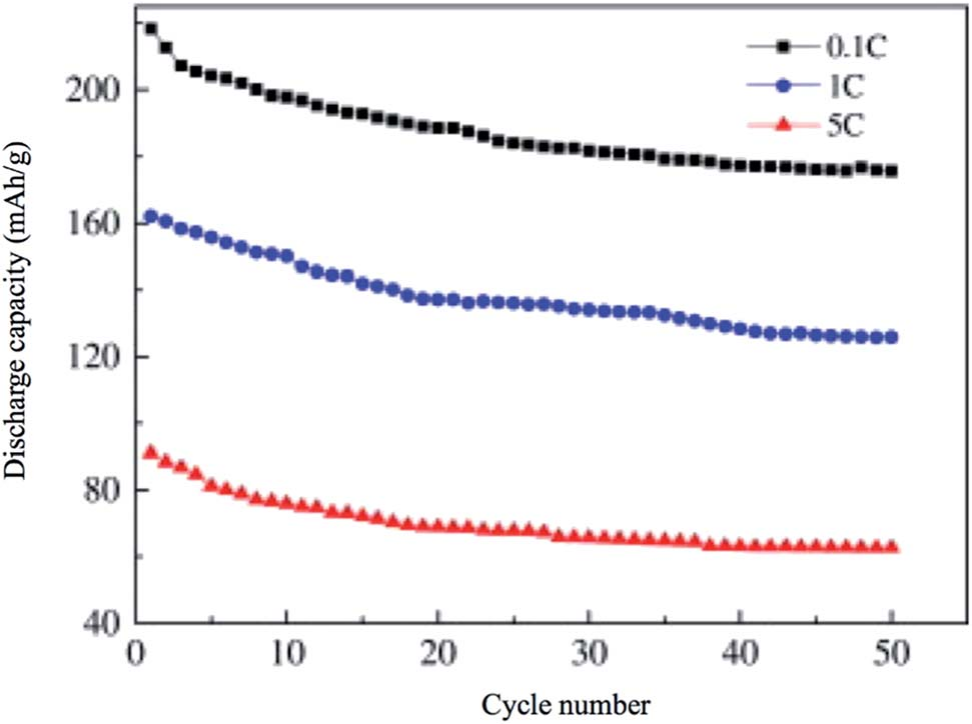
Cyclic performance of nano C/Li_2_MnSiO_4_ synthesized by ionothermal method, the material is tested in the potential range of 2.5–4.5 V.^153^

Li_2_MnSiO_4_ is a more interesting material than Li_2_FeSiO_4_ as it offers higher redox potential and specific capacity. Li_2_MnSiO_4_ possesses a theoretical capacity of 333 mA h g^−1^ because of the contribution of two Li ions to the electrochemical reaction. This is because Mn ions can be reversibly oxidized and reduced from the +2 state to +4 state with the redox couples of Mn^2+^/Mn^3+^ and Mn^3+^/Mn^4+^ at 4.1 and 4.5 V *vs.* Li^+^/Li, respectively. At 0 K, the low-temperature β-Li_3_PO_4_ is more thermodynamically stable than the high-temperature γ-Li_3_PO_4_. The transformation from β to γ structures is exothermic and can take place at higher temperatures. Also, the γ to β transformation can happen under high-pressure conditions.^142^ The separation of polymorphs is possible by applying the preferred conditions of each structure. *P*2_1_/*n* is obtained by synthesis at high temperatures, followed by fast quenching. The synthesis of *Pmn*2_1_ polymorph is possible by controlling both the pressure and temperature at 2–8 GPa and 600–900 °C, respectively.^142^ Among these polymorphs, the low-temperature orthorhombic *Pmn*2_1_ is the best polymorph as it shows favorable thermodynamic stability and exhibits the lowest Li diffusion energy barrier. Li_2_MnSiO_4_ suffers from poor performance and fast capacity fading problems upon cycling, as shown in Fig. 16. This is because of the low ionic and electronic conductivity (∼5 × 10^−16^ S cm^−1^ at RT)^143^ and the structural instability due to the presence of Mn ions in the tetrahedral sites. Several strategies were proposed to improve its structural stability upon cycling. The synthesis of new polymorphs in which the Mn ions are sitting in stable octahedral sites was tested under high pressure up to 15 GPa. However, it was not obtained because of the strong cationic repulsion in the dense structures.^144,145^ The addition of conductive carbon, such as acetylene black, showed significantly improved performance.^146^ Nano C/Li_2_MnSiO_4_ prepared by ionothermal method showed improved performance and capacity retention even at high C rates as shown in Fig. 17. Cation substitution by Mg, Ca, Cr, Ti, V, Ni, Na, Cu or P improved the intrinsic conductivity and increased the Li ion diffusion coefficient.^147–151^

## 7 Go green with Li-ion batteries

Following the growth of the Li-ion batteries industry, the environmental impact of such a technology is critical and must be carefully considered. Cathode materials are the key components in the battery in terms of cost and active weight. Their toxic byproducts during the manufacturing process as well as inadequate disposal ways, require urgent green approaches to avoid their severe health and environmental problems. One of the proposed methods in literature is to switch from conventional to green synthesis routs. To avoid environmental pollution and avert additional costs associated with processing the harmful byproducts that contain anions such as Cl^−^, SO_4_^2−^, NO_3_^−^, and gases like N_*x*_O_*y*_, CO, and NH_3_.^154^ The electrochemical performance is one of the main factors that direct the industry, therefore, considering it side by side with the environmental issues is logical and crucial. As an example, Bolloju *et al.* obtained 165 mA g^−1^ at 0.1C from LiFePO_4_ prepared using Fe powder as a precursor, which is comparable to that obtained by conventional chemical synthesis.^155^ Fe powder is very advantageous over other precursors because it does not produce harmful anions. Also, there is a 100% atom economy delivered by the iron metal, which makes it cost-effective as well. Liu *et al.* prepared multi-doped LiFePO_4_/C with Mn, Co, and Ni using Fe powder and spent electroless nickel plating solution.^156^ The product shows comparable performance to that obtained by the expensive ferrous oxalate or ferric oxide as well as to that prepared by conventional chemical routs.

**Fig. 18:**
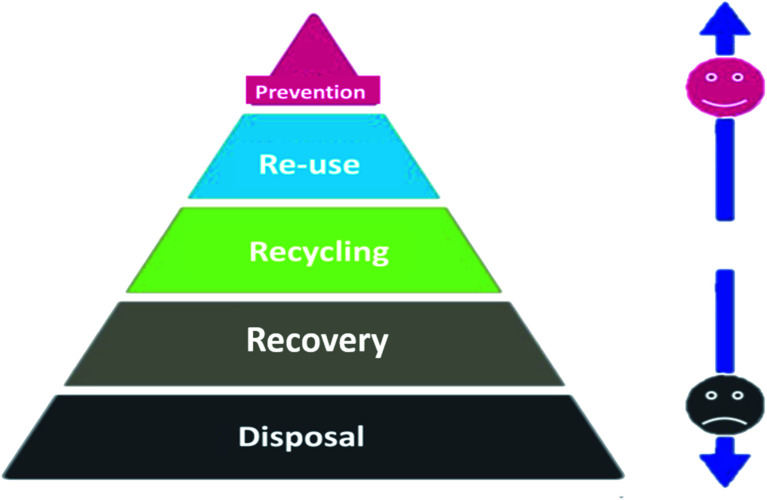
The waste management hierarchy and range of recycling options.^158^

Waste management is a process that considers the protection of the environment with various strategies in a hierarchy order (disposal, recovery, recycling, reuse, and prevention), as illustrated in Fig. 18. Accordingly, reusing a battery is cleaner than recycling because recycling needs to spend energy on transportation and processing. Thus, it is possible, for instance, to collect the batteries that retain 85% to 80% of their initial capacity from electric vehicles to reuse in stationary grid applications.^157^ Recycling can still help in decreasing pollution and satisfy the need for raw materials. Recycling relies first on the recovery of different streams of raw materials followed by a pyrometallurgical or hydrometallurgical process to extract metals (Li, Ni, Mn, Al, Cu or Co). Pyrometallurgical is simple; the battery is smelted in a high-temperature furnace to reduce the active materials into metallic alloy form and also produce slag and gases.^158^ This method requires filters to trap the emitted toxic gases.

**Fig. 19:**
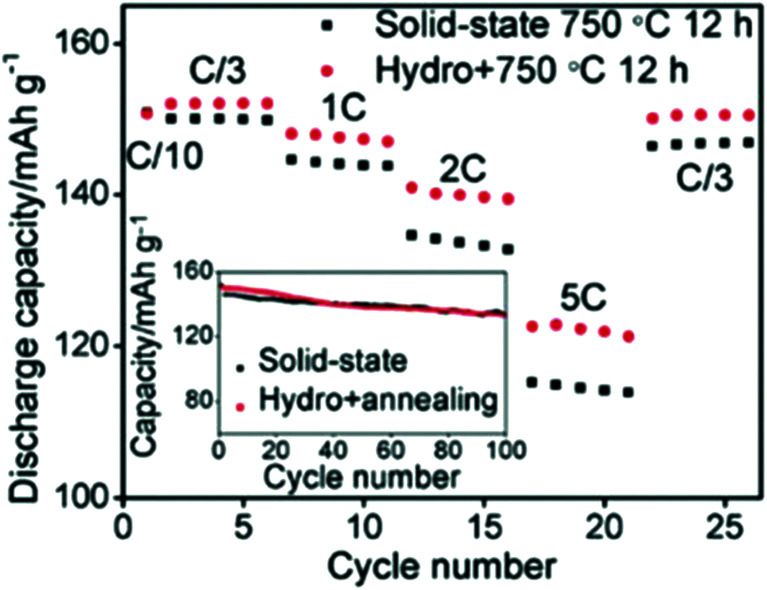
Comparison of two materials, one was sintered for 12 h at 750 °C and the other was prepared by hydrothermal method then sintered for 12 h at 750 °C.^159^

On the other hand, the hydrometallurgy process involves the leaching of the metal from the cathode by the use of aqueous solutions, such as a mixture of acid and hydrogen peroxide. Each metal can be obtained by extraction, precipitation, or electrodeposition. Shi *et al.* combined hydrothermal and annealing method to efficiently obtain LiCoO_2_ with controlled microstructure and composition and found that its rate capability is higher than that obtained by conventional solid-state synthesis (Fig. 19).^159^ Transition metals oxides and hydroxides were collected from commercial spent Li-ion batteries and used as a potential material for supercapacitors.^160,161^ The battery cathode tapes were recycled and used as a catalyst for the degradation of the toxic methylene blue dye.^162^

## 8 Summary and outlook

The use of different cathode materials, such as layered oxides, olivines, and spinels, for Li-ion batteries is summarized and discussed. The classification of these cathodes materials is based on the Li ion diffusion pathway in different structures. The principle challenge for Li-ion batteries is the development of functional materials that can offer higher energy, power, and lifetime than the currently existing materials. In order to achieve this goal, huge efforts were explored, including the development of Li-rich oxides or mixed transition metal layered oxides. Moreover, the origin of the factors affecting the battery lifetime, such as degradation of the materials upon cycling and aging, are of great importance as outlined in this review. Improving the performance of existing materials is possible through different strategies, including the creation of defects in the material, manipulation of nanostructured complexes, substitution with different transition metals, doping with foreign elements, and smart coatings. Furthermore, continued progress to safer batteries, clean green materials, and lower cost is required. To this end, modelling is a great tool to identify new cathode materials that can satisfy the balance between the electrochemical performance and environmental requirements. Moreover, recycling Li-ion batteries and recovery of their components to construct new devices should be adapted and widely investigated.

## Conflicts of interest

There are no conflicts to declare.

## Supplementary Material
